# Predictors for gram-negative monomicrobial necrotizing fasciitis in southern Taiwan

**DOI:** 10.1186/s12879-020-4796-3

**Published:** 2020-01-20

**Authors:** Tsung-Yu Huang, Kuo-Ti Peng, Cheng-Ting Hsiao, Wen-Chih Fann, Yao-Hung Tsai, Yen-Yao Li, Chien-Hui Hung, Fang-Yi Chuang, Wei-Hsiu Hsu

**Affiliations:** 1 Division of Infectious Diseases, Department of Internal Medicine, Chang Gung Memorial Hospital, Chiayi, Taiwan; 2 Graduate Institute of Clinical Medical Sciences, College of Medicine, Chang Gung University, Taoyuan, Taiwan; 3 Department of Orthopedic Surgery, Chang Gung Memorial Hospital, No. 6, West section, Chia-Pu Road, Pu-Zih city, Chiayi 61363 Taiwan; 4 Department of Medicine, School of Medicine, Chang Gung University, Tao Yuan, Taiwan; 5 Department of Emergency Medicine, Chang Gung Memorial Hospital, Chiayi, Taiwan; 6 Department of Laboratory Medicine, Chang Gung Memorial Hospital, Chiayi, Taiwan

**Keywords:** Fibrinogen, Gram-negative pathogen, Hyperlactatemia, Necrotizing fasciitis, Seafood

## Abstract

**Background:**

Necrotizing fasciitis (NF) is a rare and life-threatening necrotizing skin and soft-tissue infection. Infectious pathogens of NF must be detected early and treated rapidly to prevent loss of limb or a fatal outcome. This study aimed to detect more reliable predictors between gram-negative and gram-positive monomicrobial NF of limbs.

**Methods:**

A total of 100 patients with limb monomicrobial NF were diagnosed prospectively from April 2015 to July 2018. These monomicrobial NF pathogens can be divided into gram-negative and gram-positive groups according to the result of Gram staining and final bacterial reports. Data such as demographics, seawater or seafood contact history, infectious location, comorbidities, presenting signs and symptoms, and laboratory findings were recorded and compared.

**Results:**

A total of 55 patients were infected with gram-negative organisms and 45 patients with gram-positive organisms. Among the 55 cases of monomicrobial gram-negative NF, 48 (87.3%) were caused mainly by *Vibrio* spp. (38, 69.1%) and *Aeromonas* spp. (10, 18.2%). A higher incidence of chronic kidney disease, cerebrovascular accident, tachypnea, and septic shock; a higher rate of band forms of leukocytes of more than 3%, serum lactate of more than 20 mg/dL, and C-reactive protein level of less than 150 mg/dL; prolonged prothrombin time; and a lower fibrinogen level were observed in patients with gram-negative infection. In a multivariate analysis, a higher incidence of seawater or seafood contact history (odds ratio [OR]: 66.301; 95% confidence interval [CI]: 7.467–588.702), a higher rate of hyperlactatemia (OR: 7.904; 95% CI: 1.231–50.744), and a low fibrinogen level (OR: 1.013; 95% CI: 1.004–1.023) indicated gram-negative infection.

**Conclusions:**

In southern Taiwan, NF of limbs mainly affected the lower limbs, exhibited monomicrobial infection, and was predominated by gram-negative bacteria. Gram-negative monomicrobial NF of limbs often occurred in individuals with the more seawater or seafood contact history, hyperlactatemia, and low fibrinogen levels.

## Background

Necrotizing fasciitis (NF) is a rare and life-threatening necrotizing skin and soft-tissue infection (NSSTI) characterized by a rapid spread of necrosis in the subcutaneous tissues, particularly the superficial and deep fascia [1, 2]. Necrotizing fasciitis is associated with high mortality despite patients undergoing aggressive operative debridement and fasciotomy and adequate parenteral antibiotic therapy [2]. The overall amputation rate in NF was 4.7–22.5% [3–9], and the mortality rate was 12.1–76% [2–12]. Early fasciotomy, an appropriate antimicrobial regimen ordered with microbiologic proof, or empiric antimicrobial therapy supported by infectious-disease physicians should be performed in critically ill patients suffering from fulminant NF [13–15] to prevent loss of limb and even death.

Type I NF is a polymicrobial infection [16] that accounts for nearly 53.9–69.2% of all NF types [3, 4]. However, in Taiwan, the incidence of monomicrobial NF is reportedly 60.4–70.6% [6, 7, 12]. Chang Gung Memorial Hospital-Chiayi (CGMH-Chiayi) is a 1300-bed capacity tertiary teaching hospital situated on the western coast of southern Taiwan. Given the location, patients are exposed to occupations related to seawater or raw seafood. In our previous reports, *Vibrio* spp. and *Aeromonas* spp. have been regarded as the most important gram-negative bacteria causing NF since 2004 [8, 9, 11–13, 17–22], and *Vibrio cholerae non-O1* keratitis [23]. Our team, “Vibrio NSSTIs Treatment and Research (VTR) Group,” at CGMH-Chiayi consists of professional medical staff working in various departments, namely, emergency medicine, orthopedic surgery, infectious diseases, intensive care unit, and hyperbaric oxygen treatment center. This group has conducted considerable research focused on comparing *Vibrio vulnificus* with other different infectious microorganisms, including *Aeromonas* spp. [13], *Vibrio cholerae* non-O1 [18], and *Staphylococcus aureus* (*S. aureus*) [22].

In southwest Taiwan, Gram-negative aerobic bacteria, such as *V. vulnificus*, *A. hydrophila*, *Klebsiella pneumoniae*, and *Escherichia coli*, were the most frequently isolated microorganisms of NF [6–14, 17–22, 24–26], and they can cause more rapid, fulminant, and deadly NF than gram-positive pathogens [8, 12, 27]. In gram-positive aerobic pathogens of NF, β-hemolytic *Streptococcus* spp. and *S. aureus* were extremely important microorganisms [3, 4, 6–10, 12, 16, 22, 27–29]. In comparing gram-negative with gram-positive aerobic NF, the mortality rates of the former group were generally higher than the latter group (17.1–41.2% vs. 9.1–30.8%) [8, 12, 27]. *V. vulnificus* causes the most rapid fatal NSSTIs, with most patients having died within 48 h after admission [11, 13, 25]. From 1966 to 2014, the pooled estimate of total mortality rates about *V. vulnificus* NSSTIs from the random-effects meta-analysis was as high as 37.2% [30]. *Aeromonas* spp. are another fulminant group of microorganisms associated with high mortality of 26.7–50.0% for NF [9, 12–14, 20].

Diagnostic delays of NF are related to increased morbidity and mortality [31]. In 2011, we compared the prognostic factors and characteristics of gram-negative and gram-positive monomicrobial NF [8]. However, insights into the early detection of more reliable predictors between gram-negative and gram-positive monomicrobial NF are rarely discussed. Thus, we used a simple standard protocol that utilizes demographic data, clinical presentations, and laboratory findings to evaluate the possible pathogens of monomicrobial NF of limbs when patients arrived in the emergency department (ED) within 1 h.

## Methods

### Setting and study design

**Fig. 1:**
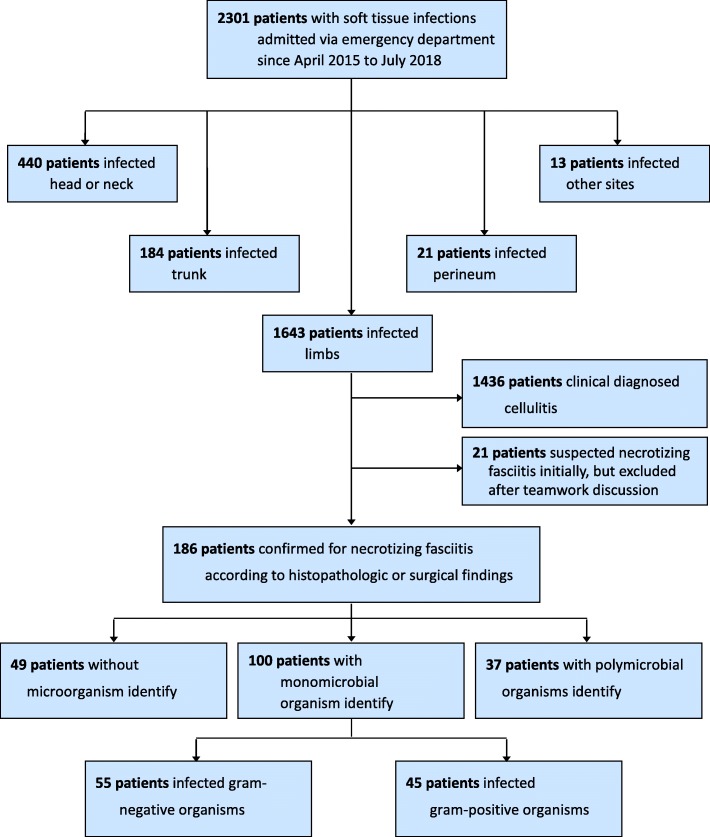
Flowchart of 100 monomicrobial necrotizing fasciitis

This is a prospective study performed by the VTR Group at CGMH-Chiayi from April 2015 to July 2018. Between these periods, these patients admitted at the ED and diagnosed with skin and soft-tissue infections were initially enrolled in our study (Fig. 1). Only monomicrobial NF of limbs could be analyzed in the study. It followed the tenets of Declaration of Helsinki. Written consent was obtained before the investigation.

Patients with monomicrobial NF of limbs were enrolled in the study using the following criteria: (1) NF was defined by surgical findings, including the presence of grayish necrotic skin, subcutaneous fat and fascia, no resistance of normally adherent fascia to digital blunt dissection, and a purulent discharge resembling foulsmelling dishwater [8, 17, 24]; (2) Histopathological tissue specimens were obtained to confirm the diagnoses [3, 32]; (3) monomicrobial infection was documented by isolating single pathogenic bacteria from soft-tissue lesions and/or blood collected immediately after the patient’s arrival at the ED or during surgery [8, 17]; and (4) such bacteria infected any limb.

### Demographic data, clinical presentations, and laboratory findings

Patients with monomicrobial NF of limbs were divided into gram-negative and gram-positive groups according to the results of Gram staining and final bacterial reports. Data such as demographics, seawater or seafood contact history, location of infection, comorbidities, presenting signs and symptoms, and laboratory findings were recorded and compared.

### Statistical analysis

The predictors for gram-negative monomicrobial NF of limbs were determined using a multivariate logistic regression model. Categorical variables were tested by Fisher’s exact test, continuous variables were tested by Student’s *t*-test or the Mann–Whitney U test, and a two-tailed *p*-value < 0.05 was considered statistically significant. Odds ratios (ORs) and 95% confidence intervals (CIs) were calculated to evaluate the strength of any association, as well as the precision of the estimated effect. All statistical calculations were performed using the Statistical Package for the Social Sciences for Windows, version 18.0 (Chicago, IL, USA).

## Results

### Patients and microbiology analysis

Between April 2015 and July 2018, 186 patients admitted via the ED were surgically confirmed to have NF of limbs (Fig. 1). Among them, 100 (53.8%) patients had a monomicrobial organism, 37 (19.9%) had polymicrobial organisms, and 49 (26.3%) had no microorganism.

The microbiological findings of these 100 monomicrobial NF cases are listed in Table 1. Gram-negative pathogens causing NF were identified in 55 (55%) patients, whereas gram-positive pathogens were detected in 45 (45%) patients. *V. vulnificus* was the most common infectious organism, accounting for 37 (37%) of the gram-negative bacillary monomicrobial NF cases, followed by the gram-negative organism *Aeromonas hydrophila*, which was isolated from 9 (9%) patients. Of the gram-positive cocci, methicillin-resistant *S. aureus* was the most commonly isolated (15%), followed by methicillin-sensitive *S. aureus* (13%) (Table 1).

**Table 1:** Summary of microbiology

Identified infectious microorganisms	Total No. (%)
Gram-negative pathogens	55 (55)
*Vibrio* spp*.*	38 (38)
*Vibrio vulnificus*	37
*Vibrio cholerae non-O1*	1
*Aeromonas* spp.	10 (10)
*Aeromonas hydrophila*	9
*Aeromonas sobria*	1
*Pseudomonas aeruginosa*	2 (2)
*Enterobacter cloacae*	2 (2)
*Shewanella putrefaciens*	1 (1)
*Escherichia coli*	1 (1)
*Klebsiella pneumoniae*	1 (1)
Gram-positive pathogens	45 (45)
*Staphylococcus* spp.	30 (30)
MRSA^a^	15
MSSA^b^	13
Coagulase-negative staphylcoccus	2
β-hemolytic *Streptococcus*	14 (14)
*Streptococcus Group Non ABD*	4
*Streptococcus pyogenes*	2
*Streptococcus agalactiae*	2
*Streptococcus equisimilis*	2
*Streptococcus dysgalactiae*	1
*Streptococcus* like	3
*Peptostreptococcus* sp	1 (1)
Total	100 (100)

*Abbreviations*: ^a^*MRSA* Methicillin-resistant *Staphylococcus aureus*, ^b^*MSSA* Methicillin-sensitive *Staphylococcus aureus*

The gram-negative group had a higher incidence of bacteremia than the gram-positive group (34/55, 61.8% vs. 8/45, 17.8%; *p* <  0.001).

### Clinical outcomes

The gram-negative group had a higher incidence of mortality rate (9/55, 16.4% vs. 3/45, 6.7%; *p* = 0.138) and amputation rate (7/55, 12.7% vs. 0/45, 0%; *p* = 0.013) than the gram-positive group.

### Surgical treatment

The gram-negative group had a higher number of debridement (2.05 ± 1.50 vs. 1.56 ± 1.34%; *p* = 0.085), amputation (0.13 ± 0.34 vs. 0 ± 0; *p* = 0.013), and fasciotomy (1.11 ± 0.42 vs. 1.04 ± 0.21; *p* = 0.345) than the gram-positive group.

### Demographic data

No significant differences in the parameters of age, gender, and infective regions were observed between these two groups. The gram-negative group was characterized by a higher incidence of seawater or seafood contact history, chronic kidney disease (CKD) and a cerebrovascular accident (CVA) (*p* <  0.001, *p* = 0.049, and *p* = 0.004, respectively; Table 2). Meanwhile, the gram-positive group was characterized by a higher incidence of gout (*p* = 0.005; Table 2).

**Table 2:** Demographic data from monomicrobial necrotizing fasciitis patients with gram-negative and gram-positive pathogens

Variable	Gram-negative pathogen (*n* = 55)	Gram-positive pathogen (*n* = 45)	*P-*value
Age (years)	68.9 ± 12.9	64.7 ± 15.4	0.107
Gender, male	37 (67.3)	32 (71.1)	0.680
Involved region			
Upper extremities	23 (41.8)	17 (37.8)	0.682
Lower extremities	32 (58.2)	28 (62.2)	0.682
Seawater or seafood contact history	38 (69.1)	7 (15.6)	< 0.001*
Underlying chronic diseases			
Alcoholism	15 (27.3)	12 (26.7)	0.946
Chronic kidney disease	18 (32.7)	7 (15.6)	0.049*
Cerebrovascular disease	14 (25.5)	2 (4.4)	0.004*
Chronic liver dysfunction			
HBV	10 (18.2)	5 (11.1)	0.325
HCV	22 (40.0)	10 (22.2)	0.058
HBV or HCV	29 (52.7)	15 (33.3)	0.052
Liver cirrhosis	17 (30.9)	10 (22.2)	0.330
Diabetes Mellitus	23 (41.8)	19 (42.2)	0.968
Gout	0 (0)	6 (13.3)	0.005*
Malignancy	11 (20.0)	7 (15.6)	0.565
Peripheral vascular disease	0 (0)	2 (4.4)	0.200

Data was presented as mean (standard deviation) or frequency (%)

*: *p*-value < 0.05

### Clinical presentations

No significant differences in the presentation of an erythematous, swollen, locally hot, and painful lesion; bulla formation; or skin necrosis were observed between these two groups (Table 3). However, the proportion of patients presenting with tachypnea (respiratory rate >  20/min, 43.6% vs. 11.1%; *p* < 0.001) and shock (systolic blood pressure < 90 mmHg, 45.5% vs. 11.1%; *p* < 0.001) was higher in the gram-negative group than in the gram-positive group (Table 3).

**Table 3:** Clinical presentations of patients with gram-negative pathogen and gram-positive pathogen associated monomicrobial necrotizing fasciitis patients

Variable	Gram-negative pathogen (*n* = 55)	Gram-positive pathogen (*n* = 45)	*P-*value
Fever (> 38 °C)	19 (34.5)	13 (28.9)	0.546
Tachycardia^a^	32 (58.2)	21 (46.7)	0.251
Tachypnea^b^	24 (43.6)	5 (11.1)	< 0.001*
Shock^c^	25 (45.5)	5 (11.1)	< 0.001*
Erythema	51 (92.7)	42 (93.3)	0.906
Swelling	51 (92.7)	41 (91.1)	0.767
Local hot	48 (87.3)	42 (93.3)	0.315
Pain or tenderness	55 (100)	45 (100)	1.000
Bullous lesions	28 (50.9)	14 (31.1)	0.066
Skin necrosis	10 (18.2)	4 (8.9)	0.183

Data was presented as mean (standard deviation) or frequency (%)

*: *p*-value < 0.05

^a^Tachycardia: heart beat > 100/min, ^b^Tachypnea: respiratory rate >  20/min, ^c^Shock: systolic blood pressure < 90 mmHg

### Laboratory findings

No significant differences in total white blood cell count, hemoglobin, platelet count, sodium, creatinine, glucose, total bilirubin, or hypoalbuminemia (serum albumin < 2.5 g/dL) were observed between the two groups (Table 4). The band forms of leukocytes of more than 3%, hyperlactatemia (serum lactate > 20.0 mg/dL), and C-reactive protein (CRP) of less than 150 mg/L were more frequently increased in the gram-negative group (*p* = 0.030, *p* < 0.001, and *p* < 0.001, respectively; Table 4). In addition, the prothrombin time (PT) values for the gram-negative group were significantly higher than those for the gram-positive group (*p* = 0.003). The proportion of patients presenting with a lower fibrinogen level was frequently observed and significantly higher in the gram-negative group (*p* < 0.001). The gram-positive group had a higher Laboratory Risk Indicator for Necrotizing Fasciitis (LRINEC) score (≥6) than the gram-negative group (62.2% vs. 32.7%, *p* = 0.003; Table 4).

**Table 4:** Laboratory findings of patients with monomicrobial necrotizing fasciitis by gram-negative pathogen and gram-positive pathogen

Variable	Gram-negative pathogen (*n* = 55)	Gram-positive pathogen (*n* = 45)	*P-*value
Total WBC^a^ count			
Leukocytosis (≧ 12,000/uL)	28 (50.9)	31 (68.9)	0.069
Leukopenia (≦ 4000/uL)	4 (7.3)	2 (4.4)	0.554
Leukocytosis or Leukopenia	32 (58.2)	33 (73.3)	0.114
Differential count			
Band forms > 3%	29 (52.7)	14 (31.1)	0.030*
Neutrophilia (> 7500/uL)	38 (69.1)	35 (77.8)	0.330
Lymphocytopenia (< 1000/uL)	34 (61.8)	23 (51.1)	0.282
Hemoglobin (< 10 g/dL)	9 (16.4)	4 (8.9)	0.269
Thrombocytopenia (< 15 × 10^4^/uL)	29 (52.7)	18 (40.0)	0.205
Hypoalbuminemia (< 2.5 g/dL)	7 (12.7)	1 (2.2)	0.054
Hyperlactatemia (> 20 mg/dL)	35 (63.6)	7 (15.6)	< 0.001*
C-reactive protein (< 150 mg/L)	40 (72.7)	17 (37.8)	< 0.001*
Creatinine (μmol/L)	180.4 ± 137.2	134.5 ± 109.7	0.072
D-dimer (mg/L)	4.3 ± 6.9	2.6 ± 5.8	0.222
Fibrinogen (mg/dL)	316.5 ± 145.8	517.4 ± 164.5	< 0.001*
Glucose (mmol/L)	9.7 ± 6.4	10.3 ± 5.2	0.647
Sodium (mmol/L)	135.5 ± 3.5	135.0 ± 3.5	0.544
LRINEC^b^ score≧6	18 (32.7)	28 (62.2)	0.003*
PT^c^ (seconds)	13.4 ± 4.9	11.0 ± 2.0	0.003*
Total bilirubin (mg/dL)	1.9 ± 1.9	1.7 ± 3.9	0.718

Data was presented as mean (standard deviation) or frequency (%)

*Abbreviations*: ^a^*WBC* White blood cell, ^b^*LRINEC* Laboratory risk indicator for necrotizing fasciitis, ^c^*PT* Prothrombin time

*: *p*-value < 0.05

### Multivariate analysis

In a multivariate analysis, a higher incidence of seawater or seafood contact history (OR: 66.301; 95% CI: 7.467–588.702; *p* < 0.001), a higher hyperlactatemia rate (OR: 7.904; 95% CI: 1.231–50.744; *p* = 0.029), and a lower fibrinogen level (OR: 1.013; 95% CI: 1.004–1.023; *p* = 0.004) were indicative of a gram-negative pathogen infection (Table 5).

**Table 5:** Multivariate regression for gram-negative pathogen from monomicrobial necrotizing fasciitis patients

	OR^a^ (95% CI^b^)	*P-*value
Seawater or seafood contact history	66.301 (7.467–588.702)	< 0.001*
Hyperlactatemia > 20 mg/dL	7.904 (1.231–50.744)	0.029*
Low fibrinogen (mg/dL)	1.013 (1.004–1.023)	0.004*
C-reactive protein < 150 mg/L	0.153 (0.013–1.863)	0.141
Tachypnea	1.273 (0.211–7.661)	0.792
Band > 3%	0.570 (0.084–3.872)	0.565
Shock	1.490 (0.177–12.566)	0.714
Chronic kidney disease	0.480 (0.075–3.052)	0.437
Cerebrovascular disease	5.601 (0.583–53.777)	0.135
PT (seconds)	0.822 (0.550–1.229)	0.339
Gout	–	0.999

*Abbreviations*: ^a^*OR* Odds ratio, ^b^*CI* Confidence interval

*: *p*-value < 0.05

## Discussion

In our study, 48 (87.3%) of the 55 patients with monomicrobial gram-negative NF were mainly infected by *Vibrio* spp. (38, 69.1%), followed by *Aeromonas* spp. (10, 18.2%). From 1988 to 1992, *V. vulnificus* infection mortality rates had induced 54.2–55.6% of patients with sepsis [25, 33] and 25.3–35.3% of patients with soft-tissue infections [25, 33]. Our VTR Group had persistently reduced the mortality rates of *Vibrio* NF from 38.5% in 2004 to 13.2% in 2012 [11–13, 17–19, 22] and of *V. vulnificus* NF from 35.3% in 2007 to 11.1% in 2012 [12, 13, 18, 19, 22]. However, the mortality rate of *Aeromonas* spp. NF had persistently increased from 26.7% in 2007 to 45.5% in 2015 [12, 13, 20]. Thus, distinguishing gram-negative from gram-positive NF is extremely important.

Having a higher incidence of seawater or seafood contact history in gram-negative NF is the most important history in our study. *Vibrio* and *Aeromonas* spp. are members of the Vibrionaceae family that can thrive in similar aquatic environments. According to past reports, approximately 53.6–100% of patients with *Vibrio* infections had a recent history of contact with seawater or raw seafood [11, 17, 18, 25, 33]. Additionally, *Aeromonas* spp. are often located in fresh or brackish water, sewage, solid, tap water, or nonfecal organic materials [13, 26, 34].

Understanding the demographic findings of patients with NF, especially chronic liver disease (hepatitis or cirrhosis of the liver), chronic alcoholism, CKD, malignant disease, diabetes mellitus, and peripheral vascular disease, is also important [4, 6–8, 11, 14, 21, 25, 27, 33]. According to previous reports, gouty arthritis was more prevalent in gram-positive NF [8, 28], and our study conforms to this result (Table 2). Gram-negative monomicrobial NF had more patients with immunocompromized disorders (CKD, diabetes mellitus, liver dysfunction, or malignancy) [8]. Compared with patients with gram-positive infections, those with gram-negative pathogens were characterized by a higher prevalence of chronic liver dysfunction [8, 12] and more likely to have a baseline malignancy [27]. We also found that the proportion of chronic liver disease and malignancy in the gram-negative group was higher than that in the gram-positive group, but it was not statistically different. This result may be due to the small number of patients. Meanwhile, patients who had NF with CKD in the gram-negative group had a higher mortality rate than those in the gram-positive group [8]. In addition, patients who had NF with CKD and CVA can easily acquire gram-negative infections (Table 2); however, this result has been less discussed in previous literature. These similar phenomena may lead to vascular sclerosis.

Furthermore, we need to check the major clinical signs and symptoms of patients with NF. Most of these patients presented with erythematous (52.3–100%) [3, 7–10], swollen (71.1–100%) [7–10], locally hot (41.1–96.6%) [3, 7, 10], painful or tender (54.7–100%) [3, 7–10], and bullous lesions (13.3–44.9%) [3, 7, 10]. In our study, the gram-negative group had a greater proportion of patients with bullous lesions (50.9% vs. 31.1%) than the gram-positive group, but it had no significant statistical difference. Hemorrhagic bullae are an important skin phenomenon of *Vibrio* infection that generally developed at the time of admission or within 24 h of hospitalization and became more severe every hour [25]. Approximately 37–46.4% of *Vibrio* infections can develop hemorrhagic bulla, especially primary *Vibrio* septicemia by 37–54.5% [25, 33] and *Vibrio* wound infection by 41–60% [25, 33]. Although bulla formation can be divided into hemorrhagic bullae or clear bullae, distinguishing them is sometimes difficult. Secondary skin lesions were found on 65.1% of patients with primary *Vibrio* septicemia [25] and can occur until 48 h later [21]. Nonetheless, the emergence of hemorrhagic bullae would be considered a feature of *Vibrio*, but it is not representative of the performance of all gram-negative bacteria. Fever (24.1–52.8%) [3, 7–10, 12, 14, 19], hypothermia (10.9–14.3%) [8, 14], tachycardia (39.8–74.2%) [3, 9], tachypnea (47.6%) [14], and shock (12.1–64.3%) [3, 7–10, 12, 14, 19] were common systemic manifestations of patients with NF. In our past study, patients with gram-negative NF are statistically more likely to have a fever than those with gram-positive NF [12]. In this study, more patients with gram-negative pathogenic infection experienced fever and tachycardia than those with gram-positive pathogenic infection, but no statistical difference was observed. As compared with NF caused by gram-positive pathogens, those with NF caused by gram-negative pathogens tended to have tachypnea and initially presented with septic shock (Table 3). Such results are consistent with those in our past reports [8, 12]. More patients presented with dyspnea in the gram-negative group than in the gram-positive group, but this result is discussed rarely in previous studies. Furthermore, more patients had circulatory infection in the gram-negative group than in the gram-positive group (61.8% vs. 17.8%). This result may be due to the idea that the gram-negative group had more septicemia-related systemic inflammatory response symptoms [35].

Moreover, we need to check specific hematologic and biochemical tests. Gram-negative pathogens are associated with a higher rate of patients with band forms of leukocytes, lymphocytopenia, or thrombocytopenia than gram-negative pathogens [8, 12]. In our study, the band forms of leukocytes of more than 3% were more common in the gram-negative group than in the gram-positive group (Table 4). CRP is synthesized primarily by the liver in response to certain proinflammatory cytokines, and it is a protein of acute systemic inflammation, thereby indicating a prime marker of inflammation. CRP is also an important laboratory parameter of the LRINEC score to distinguish NF from other soft-tissue infections; if patients had an LRINEC score greater than 6 or CRP greater than 150 mg/dL, they are diagnosed with NF [36]. However, in our study, 72.7% of patients with gram-negative NF and 37.8% of those with gram-positive NF had initial CRP values of less than 150 mg/dL on arriving at the ED, and this finding was not reported before. Lower CRP titers can confuse our judgment in diagnosing NF, especially with gram-negative infections. However, CRP can rise several days later and may decline after patients are treated. Monitoring CRP is still important, but it may not be a good tool to diagnose gram-negative NF in our study. Only 46% of patients in the gram-positive group had an LRINEC score of 6 or greater, whereas 32.7% was recorded in the gram-negative group (Table 4). Thus, the LRINEC scoring system is not applicable for determining the early management of patients with suspected *Vibrio* and *Aeromonas* NF [19], as well as in this study. In patients with NF, hyperlactatemia is independently associated with in-hospital mortality [37]. Hyperlactatemia generally occurs in patients with shock, respiratory failure, or renal failure [38], consistent with our study. Patients with NF caused by gram-negative pathogens tend to have concurrent bacteremia and initially present with septic shock [14]. Meanwhile, fibrinogen is a kind of acute-phase protein, and its blood levels rise in response to systemic inflammation, tissue injury, and certain events. Elevated fibrinogen levels in inflammation, as well as in cancer and other conditions, have been suggested to be the cause of thrombosis and vascular injury that accompany these conditions [39]. In the present study, fibrinogen consumption was faster in the gram-negative group (Table 4) than in the gram-positive group. The possible cause may be that more septicemia cases in the gram-negative group were complicated with severe tissue damage and systemic inflammation.

In a multivariate analysis, we found three important matters related to gram-negative monomicrobial NF: seawater or seafood contact history, hyperlactatemia, and low fibrinogen levels. Gathering a detailed contact history on occupational exposure to warm seawater, trauma, or seafood or freshwater fish ingestion is necessary. If NF is suspected initially, we need to check the fibrinogen and lactate levels. These two blood tests can also reflect gram-negative organisms related to sepsis.

The present study was limited by having only 100 patients in a period of over 3 years and 4 months. Another limitation is that in the present study, patients were diagnosed mainly on the basis of microbial cultures; culture-negative and polymicrobial NF were not analyzed. The third limitation was that we merely compared gram-negative NF with gram-positive NF through limb assessment.

## Conclusion

This study demonstrated the following important points: (1) In southern Taiwan, NF of limbs involved mainly the lower extremities, exhibited high monomicrobial infection, and was predominated by gram-negative bacteria; (2) a higher incidence of CKD, CVA, tachypnea, and septic shock; a higher rate of band forms of leukocytes of more than 3% and a CRP level of less than 150 mg/dL; more prolonged PT, more hyperlactatemia cases, and a lower fibrinogen level were observed in patients with gram-negative monomicrobial NF than in patients with gram-positive NF; (3) in a multivariate analysis, gram-negative monomicrobial NF of limbs often occurred in individuals with more seawater or seafood contact history, hyperlactatemia, and a low fibrinogen level.

## Data Availability

The datasets analyzed during the current study are not publicly available, due to confidentiality reasons, but are available from the corresponding author on reasonable request.

## Abbreviations

CKD: Chronic kidney disease
ED: Emergency department
LRINEC: Laboratory Risk Indicator for Necrotizing Fasciitis
NSSTI: Necrotizing skin and soft-tissue infection
